# Lecture on the Nervous Structures and the Action of the Heart

**Published:** 1858-02

**Authors:** 


*Lecture on the Nervous Structures and the Action of the Heart.*

Being one of the Lumleian Lectures read before the Royal College of Physicians, March, 1857. By Robert Lee, M. D. F. R. S.

I now proceed to relate the circumstances which led to the discovery of the ganglia and nerves of the heart, which enable us to solve the problem of the heart's motion or action. Soon after the uterine system of ganglia and nerves had been demonstrated in 1838, I began to reflect on the analogy which exists between the structure and physiology of the uterus and heart, both powerful involuntary muscular organs, yet greatly under the influence of the brain and all the organs of the body copiously supplied with nerves. It was impossible to avoid coming to the conclusion, from all the phenomena of the heart's action, that it must be endowed with a great system of ganglia and nerves, similar in some respects to those of the uterus, which had not been discovered. I felt the strongest desire to know whether, if the heart, did possess ganglia and nerves like those of the uterus, these nervous structures would be found enlarged in hypertrophy like those of the gravid uterns, and whether those of the left ventricle would be found larger than those of the right, and if the auricles were supplied more copiously than the ventricles.

The constant pulsations of the heart from the commencement to the close of life evidently required a far greater supply of nervous influence than had been represented in the works of the most celebrated anatomists. The heart acting many hours in some creatures after being cut out of their bodies: portions of it contracting rhythmically, as it is called when the heart had been cut in pieces; its movements when they had nearly ceased being roused by galvanism and mechanical irritation; the effects of stimulants and narcotics on the contractions of the heart; the circulation having been carried on in the most perfect manner through the placenta, and all the viscera of a fœtus, which I had seen born without brain and spinal cord; the symptom in cases of angina pectoris, and sudden death from organic diseases of the heart; the sympathy which exists between the heart, the lungs, stomach, liver, intestines, and above all the uterus; no explanation could be given of all these circumstances without supposing that the heart possessed great nervous centres which had never been seen, and never before suspected to exist.

I had often put this question to the most distinguished anatomists and physiologists—From what source does the heart derive its sensitive and contractile powers? but no satisfactory answer was ever received. I urged several anatomists to undertake the investigation of the nervous structures of the heart, and assured them if they did so that their labor would not be lost. At last I induced one to undertake the task. When a year had elapsed I went to see what progress had been made, and found that he was just on the point of commencing—of breaking ground; the serous membrane had not been removed from the surface of the human heart.

On the 23rd of April, 1846, an accidental circumstance occurred which made me determine to undertake the the labor myself. On that day I went into the library of the Royal Society, where I found the president, a professor of physiology, and a microscopical anatomist. A warm discussion immediately commenced respecting the nervous structures of the uterus, when it was asserted that the uterus did not require such ganglia and nerves as I had described and represented in the Philosophical Transactions, because the heart had no ganglia and few nerves. I inquired of the professor who made this assertion, if he had ever dissected the nerves of the heart, and the reply was that he had never done so; but in support of the truth of what he had affirmed, I was referred to the plates of Scarpa and of Swan, in which you see the muscular structure of the human heart is represented as almost entirely destitute of nerves, from which it might be inferred that the action of the heart did not depend upon nervous influence.

Having procured the heart of a child six years of age, that had died of disease of the brain in St. George's Hospital, and having removed all the blood by immersion in water, it was covered with strong alcohol, and on 12th September, 1846, I proceeded with these small forceps and needles, and this dissecting lens, magnifying six diameters, to see what could be discovered. In a few hours the serous membrane was removed; underneath were seen numerous branches of nerves with ganglionic enlargement wholly unconnected with the coronary arteries, ramifying on the surface, and plunging into the muscular substance of the heart. These were traced to the base, and found to terminate in a great ganglionic plexus, situated between the pulmonic artery and aorta, into which branches from the par vagum, recurrent and sympathetic, entered. The same evening I showed these ganglia to Mr. Wharton Jones, and begged him to compare them with the plates of Mr. Swan. He saw the whole surface of the left ventricle, from the base to the apex, covered with an immense plexus of ganglia and nerves. It was now obvious that the ganglia and nerves of the heart had been overlooked as much as those of the uterus, and that no argument against the existence of the ganglia and nerves of the uterus, which had been demonstrated to exist, could be drawn from the assumption that the heart had no ganglia and few nerves.

The investigation thus commenced was continued without interruption till the month of May, 1847, when I had made dissections of the ganglia and nerves of the healthy and malformed fœtal heart, of the hearts of birds, of the heart of the child at the ages of six and nine years, of the heart of the adult in the sound state, of the human heart slightly and greatly hypertrophied, and of the heart of the young and adult heifer and horse. These dissections warranted me in drawing the following general conclusions:

1. That the blood vessels and the muscular structure of the auricles and ventricles of the heart are endowed with numerous ganglia and plexuses of nerves, which have not hitherto been described or represented in the works of other anatomists.

2. That the nervous structures of the heart, which are distributed over its surface to the apex, and throughout its walls to the lining membrane and columnæ carneæ, enlarge with the natural growth of the heart before birth, during childhood and youth, until the heart has attained its full size in the adult.

3. That the ganglia and nerves of the heart enlarge like those of the gravid uterus, when the walls of the ventricles are affected with hypertrophy.

4. That the ganglia and nerves which supply the left ventricle are more than double the size of the ganglia and nerves distributed to the right side of the heart.

In prosecuting this investigation into the nervous system of the heart, I found that the great difficulty of dissecting and displaying the ganglia and nerves did not arise so much from their extreme softness, from their close and intimate connection with the blood vessels, or from the quantity of adipose matter in which they were imbedde d,as from the presence of a dense fibrous membrane or fascia, which was interposed between the serous membrane and the muscular coat, of whose existence as a distinct tissue of the heart I had no knowledge when these researches commenced. In the most recent systematic writers on anatomy, the hea.t was represented as consisting of muscular and tendinous structures, blood vessels, nerves and absorbents, enclosed between two serous membranes.

On examining this fibrous membrane after the removal of the serous covering, it is found to be possessed of great strength and firmness, glistening, semi-transparent, and resembling, in all respects, the aponeurotic expansions, or fasciæ covering muscular organs in other parts of the body. It is much stronger over the ventricles than the auricles, and it adheres so firmly where it is in immediate contact with the muscular substance of the auricles and ventricles, that its separation often cannot be effected without tearing up some of the muscular fibres to which it is attached. From the inner surface of this fascia, which I have named the Cardiac Fascia, innumerable strong fibres pass to the blood vessels, nerves, and muscular fasciculi, and adipose matter. These slender fibres, connected with or proceeding from the inner surface, accompany and surround all the blood vessels and nerves, and thay are interlaced together, so as to form a peculiar stroma, if it may be so termed, of remarkable thickness, between the fascia and all the various structures beneath which it invests and binds together in the strongest possible manner. These fibres form a complete sheath around all the arteries, veins and nerves on the surface of the heart, and accompany them as they dip down between the muscular fasciculi, to which these branches are distributed threughout the entire walls of the heart, from the surface to the lining membrane. The cardiac fascia is obviously one of the principal causes of the firmness and strength of the central organ of the circulation of the blood, as it binds together into one mass, and gives support to the muscular fibres, like the fasciæ investing other muscles. The thin serous covering of the hear can possess little power, and add nothing to the strength of the parietes, and probably but for the fascia now described, the heart would often yield in all directions.

In a pathological point of view, the cardiac fascia is perhaps not less worthy of notice. Muscular structure, it is well known, is not liable to attacks either of common or specific inflammation. It is impossible to avoid suspecting that rheumatic inflammation of the heart has for its principal seat this dense fibrous membrane lying between the serous and muscular coats of the heart, and that attacks of rheematism of the heart do not commence primarily in the muscular structure. The tunica sclerotica of the eye sometimes becomes inflamed, softens and yields; and from these changes it is known that sclerotic staphyloma and other diseases are the results. Whether in dilatation of the heart a similar morbid change is not first set up in the fascia, and what influence this fibrous membrane has in modifying all the diseases of the heart, future observations must determine.

After the removal of the serous membrane from the surface of the ventricles, there are plexuses of ganglionic nerves readily seen with the naked eye through the cardiac fascia, ramifying on the muscular substance of the heart. If these superficial nerves, situated immediately under the cardiac fascia, be traced backward towards the base of the ventricles, they are found to terminate in a great ganglionic plexus, situated between the pulmonary artery and aorta, to the outer coat of which it adheres much more firmly than to the pulmonary artery. This is the nervous plexus between the pulmonary artery and aorta described by Falopius more than three hundred years ago. Into this great nervous mass, which enlarges as it passes to the base of the ventricles, branches of nerves enter from the par vagum, recurrent, and sympathetic nervss. From the par vagum or recurrent and great sympathetic, branches pass to the heart behind the aorta or pulmonary artery; but the great ganglionic mass of nerves situated between the aorta and pulmonary artery is properly the root of all the cardiac nerves and ganglia. From the right side of the ganglionic mass, several broad, flat branches of nerves, invested with a soft neurilemma, and accompanied by small blood-vessels, proceed to the right auricle, right ventricle, and to the septum between the ventricles. From the left side of this nervous mass, under the arch of the aorta, several large, flat nerves, likewise enveloped in a neurilemma, and accompanied by small blood vessels, proceed to the left auricle, left ventricle, and the inter-ventricular septnm. These large, flat nerves pass to the root of the left coronary artery, which they not only completely surround like a sheath, but likewise cover a portion of the aorta near its origin. Many large branches of nerves with ganglia formed upon them, accompany not only all the branches of the coronary arteries to the apex, but all the branches which pass into the muscular substance of the ventricle, and are distributed throughout its walls to the lining membrane and columnæ carneæ. From the deep nerves and ganglia of the ventricles, the muscular structure is chiefly supplied. From the great mass of nerves situated around the roots of the coronary arteries and the aorta, there are numerous branches of nerves with ganglia distributed over the muscular walls of the ventricles of the human heart, which do not accompany the blood-vessels.

On the portions of the ventricles which are devoid of fat, those ganglia and nerves are distinctly visible to the naked eye, through the serous membrane and cardiac fascia, and present a very remarkable appearance. These superficial cardiac nerves are remarkably soft, flat, and somewhat semi-transparent, as Scarpa has described, with a grey color, and the smaller branches are enveloped in a soft sheath or neurilemma, which has been regarded by all anatomists as an essential constituent tissue of the nervous structures. Towards the left side and apex of the left ventricle, these nerves lie in grooves or depressions in the muscular substance, and they spread out into ganglionic enlargements, from which laterally innumerable small filaments are sent off to the coats of the blood vessels which sink deep into the substance of the heart. It can clearly be demonstrated that every artery distributed throughout the walls of the uterus and heart, and every muscular fasciculus of these organs, is supplied with nerves upon which ganglia are formed, and which are the sources of all their contractile powers.

The preparations of the ganglia and nerves of the heart from which this description has been drawn, and the engravings from the Philosophical Transactions, are now placed before you. With the dissecting lens the ganglia and nerves are far more distinctly seen than with the naked eye. There is a very correct drawing of the ganglia and nerves of the heart of the race-horse, not engraved. (Placed also upon the table.)

This anatomical demonstration of the ganglia and nerves of the muscular substance of the heart completely overthrows the last remaining argument employed by those physiologists who still defend the doctrine that the irritability and contractility of muscular fibre are independent of nervous influence. This demonstration of the existence of numerous ganglia on the surface and throughout the walls of the heart, further clearly indicates the source of the actions of the heart as an entire organ, and how its detached parts can continue to contract after its total separation from the body. It likewise furnishes a satisfactory explanation of all the phenomena observed in the progress of all the functional and organic diseases of the heart.

The circulation of the blood could not have been carried on and life maintained, if the auricles and ventricles of the heart had not alternately contracted and relaxed as they do. Before these ganglia and nerves of the heart were demonstrated, it was impossible to explain the cause of this action or motion of the heart. By some this has been referred to the influence of the brain or spinal cord, while others believed that the contractions of the heart did not depend upon nervous influence. It must now be evident that there is nothing in the action of the heart more mysterious than in the action of the diaphragm and intercostal muscles, and all the muscles connected with respiration and digestion, the larynx, pharynx, œsophagus, stomach, intestines, bladder, uterus, and all the other voluntary and involuntary muscles of the body; the sensitive and contractile powers of the whole muscular system being derived from the nervous structures with which they are endowed, and which are all intimately connected with the brain and spinal cord,—constitute, in fact, one great system.

